# Ribosome Biogenesis and Cancer: Overview on Ribosomal Proteins

**DOI:** 10.3390/ijms22115496

**Published:** 2021-05-23

**Authors:** Annalisa Pecoraro, Martina Pagano, Giulia Russo, Annapina Russo

**Affiliations:** Department of Pharmacy, University of Naples “Federico II”, Via Domenico Montesano 49, 80131 Naples, Italy; annalisa.pecoraro@unina.it (A.P.); martina.pag1989@gmail.com (M.P.)

**Keywords:** ribosome biogenesis, mitoribosome biogenesis, ribosomal proteins, mitochondrial ribosomal proteins, cancer, cancer treatment

## Abstract

Cytosolic ribosomes (cytoribosomes) are macromolecular ribonucleoprotein complexes that are assembled from ribosomal RNA and ribosomal proteins, which are essential for protein biosynthesis. Mitochondrial ribosomes (mitoribosomes) perform translation of the proteins essential for the oxidative phosphorylation system. The biogenesis of cytoribosomes and mitoribosomes includes ribosomal RNA processing, modification and binding to ribosomal proteins and is assisted by numerous biogenesis factors. This is a major energy-consuming process in the cell and, therefore, is highly coordinated and sensitive to several cellular stressors. In mitochondria, the regulation of mitoribosome biogenesis is essential for cellular respiration, a process linked to cell growth and proliferation. This review briefly overviews the key stages of cytosolic and mitochondrial ribosome biogenesis; summarizes the main steps of ribosome biogenesis alterations occurring during tumorigenesis, highlighting the changes in the expression level of cytosolic ribosomal proteins (CRPs) and mitochondrial ribosomal proteins (MRPs) in different types of tumors; focuses on the currently available information regarding the extra-ribosomal functions of CRPs and MRPs correlated to cancer; and discusses the role of CRPs and MRPs as biomarkers and/or molecular targets in cancer treatment.

## 1. Introduction

Ribosome biogenesis is an energy-consuming and well-orchestrated process, requiring several assembly and maturation factors that are strictly regulated by different cellular inputs such as mitogenic signals and nutrient availability [1]. The ribosome is a structurally and functionally conserved supramolecular ribonucleoprotein (RNP) complex essential for the translation of information contained in messenger RNAs (mRNAs) into functional proteins, the ultimate step in the genetic program [1].

In eukaryotic cells, ribosomes are present in the cytoplasm and within semiautonomous eukaryotic organelles, mitochondria and chloroplasts [1]. Given the fact that protein biosynthesis is directly coupled with several pathways and depends on ribosome production, it is not surprising that ribosome biogenesis plays a crucial role in the orchestration of major cellular processes.

In this review, we attempt to offer an overview of cytosolic and mitochondrial ribosome biogenesis in health and cancer, focusing on the role of some cytosolic and mitochondrial ribosomal proteins in tumorigenesis.

## 2. Ribosome Biogenesis in Health

### 2.1. Cytosolic Ribosome Biogenesis

In eukaryotic cells, the cytosolic 80S ribosome is composed of two subunits: the small 40S subunit and the large 60S subunit. The 40S subunit, consisting of the 18S ribosomal RNA (rRNA) and 33 cytosolic ribosomal proteins (CRPs), decodes mRNAs by aminoacyl–transfer RNA (tRNA), whereas the 60S subunit, composed of 5S, 5.8S, and 28S rRNAs and 47 CRPs, catalyzes peptide bond formation by the peptidyl-transferase reaction [1]. The production of ribosomes appears to be a process of extraordinary complexity taking place within the nucleolus, a well-characterized subnuclear compartment without a membrane. This process engages a large number of molecular players such as RNA polymerases (RNA Pol), rRNAs, small nucleolar RNAs (snoRNAs), CRPs, regulatory, processing, assembling and maturation factors that direct the hierarchical assembly of CRPs and several rRNA folding steps [2]. Briefly, the assembly of 90S pre-ribosomes occurs in the nucleolus. Then, it will be modified and separated into pre-60S and pre-40S particles. These two pre-ribosomal particles mature along independent pathways by transiently interacting with several assembly factors (AFs) and are separately transported in the cytoplasm. After the acquisition of export competences, including the shielding of the negatively charged surface of pre-ribosomes, the exit of pre-ribosomal particles through the hydrophobic nuclear pore complexes occurs in a CMR1 and GTP binding nuclear protein-Ran (Ran–GTP)-dependent manner. In the cytoplasm, they mature to 60S and 40S subunits to form the final 80S ribosome [2,3].

### 2.2. Pre-rRNA Synthesis and Maturation

**Figure 1:**
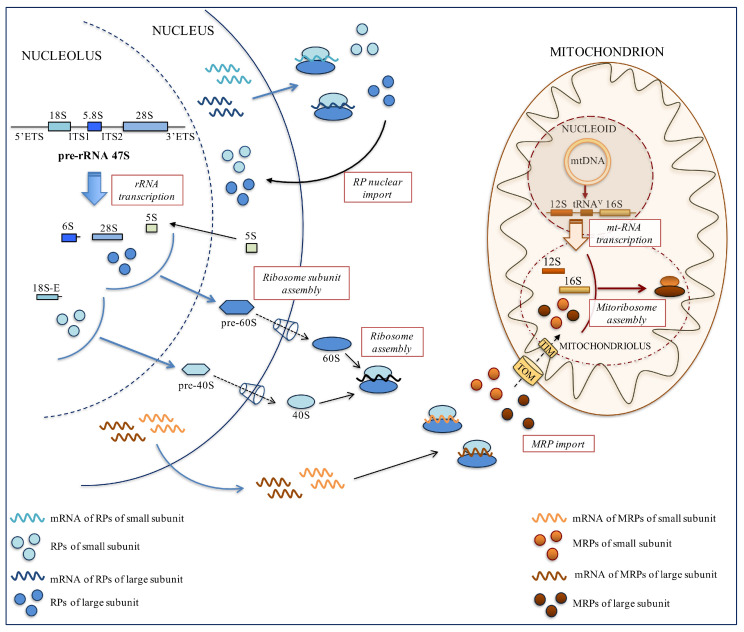
Schematic representation of cytosolic and mitochondrial ribosome biogenesis.

rRNAs, the scaffold of the ribosomal subunits and the hub of the catalytic activities of the ribosome are produced from a long primary transcript, called 47S pre-rRNA. RNA Pol I drives the transcription of ribosomal DNA (rDNA) genes, located in the nucleolar organizer regions (NORs), into the nucleoli. The primary 47S pre-rRNA transcript is characterized by the presence of the 5′ and 3′ external transcribed spacers (ETS) and two internal transcribed spacers (ITS1 and ITS2), which delimit the 18S, 5.8S and 28S rRNAs (Figure 1). These transcribed spacers contain multiple cleavage sites targeted by endo- and exonucleases, leading to the generation of mature rRNAs [4]. The initial cleavage at two distinct sites, located in the 5′ and 3′ ETS, produces the 45S pre-rRNA from which the 30S and 43S pre-rRNAs are generated. The further processing of 30S pre-rRNA results in the formation of 18S-E precursor, whereas the multiple cleavages of 43S pre-rRNA give rise to 5.8S precursor (6S pre-rRNA) and mature 28S rRNAs [3,4] (Figure 1).

Apart from the removal of the transcribed spacers, the pre-rRNAs undergo nucleotide modifications; among them emerge pseudouridylation and 2′-O-ribose methylation performed by two families of small nucleolar RNPs (snoRNPs), respectively known as H/ACA box and C/D box snoRNPs. Cleavages and chemical modifications take place simultaneously to the RNA folding steps, including successive maturation of rRNA expansion segments and the stabilization of subdomains of pre-rRNAs and their assembly with CRPs [4].

Transcription of the 5S rRNA, unlike the 47S pre-rRNA, is driven by RNA pol III in the nucleus and requires a specific regulatory factor called transcription factor IIIA (TFIIIA). Hence, the association of TFIIIA with the other general class III initiation factors, TFIIIB and TFIIIC, on the 5S gene promoter induces its transcription [4].

In the cytoplasm, the rRNA precursor 18S-E is cleaved at site 3 by the endonuclease NOB1 to generate mature 18S [3]. Similarly, the final trimming step in 5.8S rRNA processing is likely catalyzed by exonuclease ERI1, as shown in mouse [4]. It has been reported that this 5.8S rRNA final maturation step takes place in the cytoplasm in yeast and *Xenopus laevis*. However, it has not yet been demonstrated in mammalian cells [4].

### 2.3. Nucleolar Assembly of pre-60S and 40S Subunits

A key step for ribosome assembly is to incorporate CRPs onto dynamically folding pre-rRNA. After the transcription of CRP genes by RNA pol II and the synthesis of RPs in the cytoplasm, CRPs are imported into the nucleus, where they assemble with the nascent pre-rRNAs to form pre-ribosomal particles; few CRPs (e.g., stalk proteins) are assembled in the cytoplasm (Figure 1). After translation, the rapid import of CRPs in the nucleus is crucial, since unassembled CRPs are toxic to the cell and rapidly degraded [2,5]. Our group has demonstrated that mRNAs coding for CRPs are directed to the perinuclear region by using localization elements contained in their 3′ untranslated region and translated on polysomes anchored to this region. This selective localization contributes to the rapid and efficient import of newly synthesized CRPs in the nucleus [6,7,8] (Figure 1). The nuclear and the nucleolar import of CRPs is facilitated by transporters that recognize their nuclear or nucleolar localization signals, respectively [6,7,8,9]. The efficient assembly of newly synthesized CRPs into nascent ribosomes is also ensured by a class of proteins known as dedicated CRP chaperones. These proteins contribute to protecting newly synthesized CRPs from aggregation and degradation [10,11,12,13,14,15,16] (Table 1).

All these dedicated CRP chaperones accompany CRPs on their way to assembly into ribosomal subunits. The newly formed pre-ribosomal particles, pre-40S and pre-60S, are then exported in the cytoplasm where they are further processed (Figure 1). In particular, few CRPs are thought to stably assemble in the cytoplasm, including uL10 (P0), uL16, eL24 (L24), eL40 (L40), uS3, eS10 (S10) and eS26 [17].

Some CRPs play a key role in the final ribosome subunit structures and functions. For example, the functionality of eukaryotic ribosomes is ensured by the correct structure of specific bridges. The yeast ribosome contains five bridges. Among these, the bridge eB13 is important for correct subunit joining during the initiation and elongation steps of protein biosynthesis. The functional integrity of this bridge depends on multiple protein–protein and rRNA–protein interactions such as those of eL24 and uL3 with eS6 and 18S rRNA [18].

uL3 is also essential for the formation of early pre-60S particles and for the structural organization and activity of the peptidyl transferase center (PTC) [17]. uL3 conforms as a 3-fingered structure composed of a globular domain and the three extensions—the N-terminal extension, the “tryptophan finger” (finger W) and the “base thumb”—that extend into the central core of the large subunit close to PTC. Mutation studies have shown that these regions are crucial for the efficient function of the ribosome [19].

### 2.4. Mitochondrial Ribosome Biogenesis

In eukaryotic cells, mitoribosomes represent a distinct class of ribosomes that reside in the matrix of the mitochondrion and play a key role in regulating cellular respiration.

The mitochondrial 55S ribosome is composed of two subunits: the large 39S subunit, which is involved in catalyzing the peptidyl-transferase reaction, and the small 28S subunit which provides the platform for mRNA binding and decoding. The 39S subunit is composed of 16S mitochondrial rRNA (mt-rRNAs), and 50 mitoribosomal proteins (MRPs), whereas the 28S subunit consists of 12S mt-rRNAs and 29 MRPs [20].

In humans, the mitochondrial DNA (mtDNA) encodes 13 proteins, 2 mt-rRNAs (12S and 16S mt-rRNAs) and 22 tRNAs [21]. 12S and 16S mt-rRNAs are transcribed from mtDNA genes by a single subunit bacteriophage-related RNA polymerase (POLRMT) as the mt-rRNA precursor. The core machinery involved in this process includes the high mobility group box DNA-binding protein h-mtTFA/TFAM, and two transcriptional co-activator proteins, h-mtTFB1 and h-mtTFB2. Specifically, the h-mtTFB factors interact with h-mtTFA to establish a bridge between the promoter-bound h-mtTFA and POLRMT, promoting transcription initiation [21]. The mt-rRNA precursor containing 12S-tRNA^V^-16S-RNA is cleaved by RNase P and the endonuclease ELAC2 to form mature 12S and 16S mt-rRNAs [21] (Figure 1).

Mitoribosome biogenesis takes place in specific compartments within the mitochondrial matrix near the mtDNA nucleoid termed RNA granule or mitochondriolus. This compartment contains RNA processing enzymes, MRPs and other proteins required for mitoribosome biogenesis [22]. This process can be described as a strictly regulated and well-defined hierarchical mechanism in which each step is driven by the cooperation of several proteins and RNA molecules and in which the expression of both a nuclear and mitochondrial genome is necessary. The MRPs are encoded by the nuclear genome; once transcribed, the corresponding mRNAs are transported on the cytoskeleton to localize on cytoribosomes in the proximity of the mitochondria and then, the nascent MRPs are imported into the organelle [23]. This process corroborates the efficient import of MRPs, through the translocase of the outer membrane (TOM) and translocase of the inner membrane (TIM), into the mitochondrial matrix (Figure 1). Unassembled copies of MRPs, not actively involved in mitoribosome assembly, are degraded to avoid excessive accumulation in the organelle [21].

The last step in mitoribosome biogenesis, i.e., maturation of each subunit and the assembly of the mature subunits into functional mitoribosomes, still remains largely underexplored.

Recently, analysis of the structure of some assembly intermediates of large and small mitoribosomal subunits (LSU and SSU) of the human parasite *T. brucei* provided insight into this process [24]. Analysis of these structures indicates that the assembly of mitoribosomes occurs through an extensive protein network formed by assembly factors and several GTPases, which ensures the remodeling of RNA structure and RNA–protein contacts.

In particular, Jaskolowski et al. identified the structure of two late-stage assembly intermediates (state A and B). Both complexes lack 22 and 17 MRPs, respectively, out of the 72 found in the mature LSUs [25].

During conversion from intermediate state A to B, at least nine assembly factors of state A are replaced by four MRPs (mL90, mL99, mL100, and mL101) and five new assembly factors. These changes are also associated with conformational changes in the 12S rRNA and MRPs.

Progressive maturation of assembly intermediate state B to the mature LSU likely includes multiple steps and intermediates, since all the assembly factors must leave such that the 12S rRNA core, including the PTC, can adopt its mature conformation [25].

Three assembly intermediates of increasing complexity and size from the *T. brucei* SSU have been also identified [24].

The transition between three assembly intermediates and the mature SSU involves specific maturation steps including the formation of the central pseudoknot (CPK) and the helix h18 after the head, body, and platform have been largely preassembled [26].

## 3. Role of Ribosome Biogenesis in Neoplastic Transformation

Tumor cells are characterized by a higher production of ribosomes, necessary to sustain enhanced growth and subsequent cell division. The increase in ribosome production is associated with aberrant ribosome biogenesis homeostasis and alteration in number, size and shape of nucleoli; these elements represent specific hallmarks of cancer cells [27]. Furthermore, a growing amount of evidence indicates the existence of a strong relationship between the alteration in rRNA synthesis, the deregulation of some RPs—either mitochondrial or cytosolic—and the development of human cancers [27]. In particular, accumulating evidence indicates that altered expression of an individual RP may impact ribosome biogenesis, causing a condition known as nucleolar or ribosomal stress. In response to this stress, several CRPs as ribosome free forms translocate to the nucleoplasm to exert extra-ribosomal functions. Some of these free CRPs regulate the p53 mouse double minute 2 homolog (MDM2) pathway; other CRPs act through different pathways independently of p53 [28]. uL18 and uL5 have a crucial role in the p53-dependent nucleolar stress signaling pathway, which is activated in conditions of altered ribosome biogenesis [28]. In particular, it has been demonstrated that uL18 and uL5 regulate p53 homeostasis and its activation together with 5S rRNA as part of a ribosomal subcomplex—the 5S ribonucleoprotein particle (5S RNP) [29,30]. Specifically, in stressed cells, uL5 translocates into the nucleoplasm and associated with the pre-existing 5S/uL18 complex to form 5S RNP that binds to MDM2, thus regulating p53 levels [31]. To date, the 5S RNP-MDM2 pathway is thought to be implicated in the response to nucleolar stress induced by chemotherapeutic drugs, nutrient starvation, overexpression of tumor suppressor p14ARF, and also to different forms of stress, including hypoxia and oxidative stress. Furthermore, 5S RNP is also involved in the induction of a p53-mediated cellular senescence in response to oncogenic and replicative stress [31].

**Figure 2:**
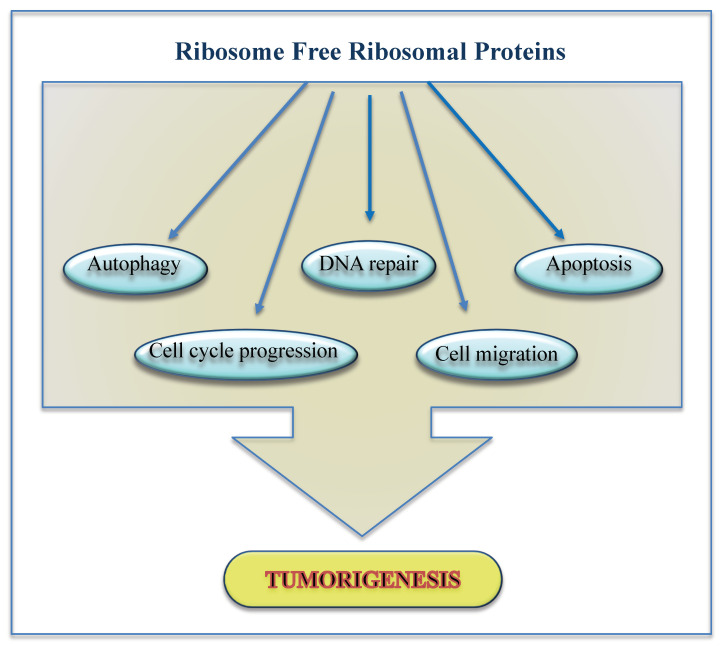
Role of ribosome free RPs in cancer.

Overall, the extra-ribosomal function of RPs regulates diverse cellular processes including cell cycle, DNA repair, maintenance of genome integrity, cellular proliferation, apoptosis, autophagy, cell migration and invasion (Figure 2) [27,28,32]. Furthermore, data from our group have demonstrated that uL3 autoregulates its own expression [33,34] and uL3 status is essential for cell response to certain anticancer drugs in p53 mutated lung and p53 deleted colon cancer cells. In particular, uL3 downregulation positively correlates with multidrug resistance. This protein influences p21 activity independently of p53 in response to nucleolar stress induced by anticancer treatments [35,36,37].

In most cases, the extra-ribosomal functions of RPs have been identified by siRNA-based knockdown approaches. Of course, this experimental strategy also affects the rate of ribosome biogenesis that needs to be upregulated in cancer cells. Moreover, altered expression of a single RP may also alter the ribosome’s translational efficiency of specific mRNAs including transcripts of other RPs or transcripts involved in the key regulatory steps of tumorigenesis and drug resistance [38,39,40]. In this scenario, it cannot be excluded that the observed effects could also be a consequence of a defect in the rate of ribosome biogenesis and/or of altered activity of ribosomes.

### 3.1. Alteration of rRNA Synthesis

RNA Pol I represents a convergence point of all information from cellular signaling cascades. In the past few decades, different studies have demonstrated that the hyperactivation of oncogenes or the inactivation of tumor suppressors found in many tumors stimulate RNA Pol I transcription, leading to the upregulation of rRNA synthesis with a consequent increase in cell growth and proliferation [41].

In many types of tumors, the constitutive activation of kinase pathways including mitogen-activated protein kinase (MAPK)/extracellular signal-regulated kinase (ERK), phosphatidylinositol 3-kinase (PI3K)/protein kinase B (Akt), mammalian target of rapamycin (mTOR), and casein kinase II (CK2) is associated with the strong activation of RNA Pol I [42].

Several studies have revealed that different kinds of cancer mutated of retinoblastoma protein (RB) and p53 show the upregulation of rRNA synthesis and more aggressive phenotype in comparison with those non-mutated in RB and p53 [42]. Specifically, phosphorylated RB (pRB) has a crucial role in controlling ribosome biogenesis. It has been demonstrated that pRB binds to and may recruit histone deacetylases (HDAC) to rDNA, causing the inhibition of rDNA transcription. In addition, other studies have shown that pRB can directly bind the upstream binding factor (UBF), a trans-acting factor required for efficient transcription driven by RNA Pol I. This interaction could either reduce the DNA-binding affinity of UBF or prevent the interaction between selective factor 1 (SL1) and UBF, resulting in the inhibition of rDNA transcription in vitro and in vivo [42].

The tumor suppressor p53, as pRB, negatively regulates cell cycle progression and directly counteracts ribosome biogenesis. In particular, p53 binds to SL1, preventing its interaction with SL1 with consequent inhibition of rDNA transcription [42].

Another important tumor suppressor, whose function is commonly lost in human cancers, is phosphatase and tensin homolog (PTEN). It has been demonstrated that PTEN suppresses RNA Pol I transcription in different human cell lines and this activity requires its lipid phosphatase activity and is independent of the p53 status [43].

The proto-oncoprotein c-Myc, mostly upregulated in tumors, represents a crucial regulator of either cytoribosome or mitoribosome biogenesis. Among different functions, the tumor-promoting activity of the c-Myc is partially due to its ability to positively regulate the nucleolar RNA Pol I and the mitochondrial POLRMT. In the nucleolus, c-Myc induces the hyperacetylation of histones and transcriptional activation of RNA Pol I, interacting with SL1 or directly with the E-box target sequence of rDNA [44,45]. At the nuclear level, c-Myc, concomitantly, can also induce mitoribosome synthesis by upregulating RNA Pol II-mediated transcription of POLRMT and other MRPs such as uS5m (MRPS5) and mS27 (MRPS27) [45]. In addition, some evidence indicates that c-Myc expression and activity are regulated by some of its downstream transcriptional target genes that encode CRPs as uL18 and uL5. uL18 cooperates with uL5 in inhibiting the expression of c-Myc through a RISC-mediated miRNA targeting mechanism [46].

All these studies suggest that the alteration of gene transcription driven by RNA Pol I and/or POLRMT, through different mechanisms, may promote neoplastic transformation. Thus, tumor cells become addicted to a high level of rRNA and/or mt-rRNA synthesis, resulting susceptible to RNA Pol I and/or POLRMT inhibition.

### 3.2. Ribosome Free RP Expression Regulate Cell Cycle

Different studies have established a connection between RPs and cell cycle progression; in fact, the deregulation of several RPs in tumors has been related to alteration in cell growth and transformation. RPs control the progression of the cell cycle, acting on families of proteins with specific functions in each phase of the cell cycle (Table 2). Some RPs modulate positive cell cycle regulators such as cyclins or cyclin-dependent kinases (CDKs), heterodimeric protein kinases that coordinate cell cycle progression through phosphorylation [27].

For example, uL10m regulates the activity of Cyclin B1/CDK1, a key player in cell cycle transition from late G2 to mitosis. Recent data have identified some components of the mitochondrial electron transport chain as potential Cyclin B1/CDK1 phosphorylation targets. Cyclin B1/CDK1 can enhance mitochondrial ATP generation via phosphorylation of its targets in mitochondrial complex I, which provides energy for cell cycle progression. Silencing of uL10m downregulates CDK1 kinase activity and leads to mitochondrial fusion via dephosphorylation of Dynamin-related protein 1 (Drp1) at Serine 616 [47].

Other RPs can indirectly modulate the cell cycle, acting on the expression and/or the activity of other modulators involved in the regulation of the cell cycle as cyclin-dependent kinase inhibitors (CKI) p21 and p27 [32].

To date, uL3 induces G1 cell cycle arrest through the formation of protein complex including nucleophosmin (NPM) and specificity protein 1 (SP1) that is involved in the regulation of p21 expression and stability [48,49]. uS15 inhibits p27 mRNA expression, accelerating G1/S transition and promoting the cell growth and cell cycle progression of gastric cancer cells [50].

uS8, a highly conserved CRP associated with 40S, is critical for cell growth and proliferation. uS8 was found widely expressed in different human colorectal cell lines and its knockdown strongly induced cell growth suppression and cell cycle arrest through p21 upregulation and CDK1 downregulation [51].

Beyond cyclins and CDKs, other important effectors play a key role in regulating the cell cycle such as Akt and E2F Transcription Factor 1 (E2F1). The knockdown of uS8 strongly reduces the phosphorylated level of Akt (pAkt) with consequent G0/G1 cell cycle arrest [52], whereas eL21 controls G1/S phase progression through the regulation of E2F1 transcription factor and E2F1 target genes [53].

Chen et al. have demonstrated that MRPS36 delays cell cycle progression and proliferation by altering the expression and post-translational modification of p53 and its target p21. Specifically, MRPS36 phosphorylates p53 at Serine 15, stabilizing it [54].

Furthermore, it has been shown that mL41 stabilizes p53 and enhances its transport to the mitochondria, thus activating apoptosis. Of interest, in cells devoid of p53, mL41 stabilizes p27^Kip1^ and induces cell cycle arrest at the G1 phase [55].

### 3.3. Ribosome Free RPs Take Part to DNA Repair

In the last few decades, many studies have demonstrated a role in DNA repair for different RPs. To date, uS3 is involved in the base excision repair (BER) pathway. Upon genotoxic stress, uS3 is phosphorylated at Threonine residue (T42) by ERK1/2 and then translocates from the cytoribosomes to the nucleus. Immunofluorescence microscopy analysis revealed that uS3 colocalizes to foci of 7, 8-dihydro-8- oxoguanine (8-oxoG) [56] and increases the glycosylase activity of 8-oxoguanine DNA glycosylase in the elimination of DNA lesions [57].

A strong correlation between intracellular levels of uL3 and the activity of specific DNA repair processes such as homologous recombination (HR) and non-homologous end-joining (NHEJ) has been demonstrated by our group, implying a significant role for uL3 in the regulation of the DNA repair process. In particular, uL3 exhibits a strong effect in inhibiting the precise NHEJ known as classical end-joining [58].

Recently, Yang et al. have identified eL6 (L6) as a crucial regulatory factor involved in DNA damage response. The authors demonstrated that eL6 is recruited to DNA damage sites in a poly-(ADP-ribose) polymerase (PARP)-dependent manner, through its interaction with histone H2A. Interestingly, other CRPs such as uL2 (L8) and uS11 are also recruited to DNA damage sites along with eL6. The silencing of eL6 inhibits the binding of the mediator of DNA damage checkpoint 1 (MDC1) and H2A histone family member X (γH2AX) to DNA damage sites, and reduces H2A/H2AX ubiquitination. Consequently, eL6 silencing results in defects in the DNA damage-induced G2/M checkpoint, DNA damage repair and cell survival [59].

Furthermore, it has been reported that MDM2 specifically binds telomere maintenance protein nibrin (NBS1), a component of the MRE11/RAD50/NBS1 (MRN) DNA repair complex, and significantly endangers genomic stability. Interestingly, the NBS1-binding region on MDM2 (amino acid 198–228) overlaps with the binding site for some CRPs including uL14, uL24 (L26), and uS11. Thus, the maintenance of genomic stability is likely due to the masking of the NBS1 binding region on MDM2 by these RPs [60,61]. However, the potential role of these CRPs as genomic instability sensors needs to be further experimentally investigated.

In addition, eS26 participates in the DNA repair process by directly modulating p53 transcriptional activity in response to DNA damage. In eS26 -depleted cells, DNA damage can still induce p53 accumulation, but p53 failed to be acetylated and efficiently transactivate its target genes [62].

Finally, a role of RPS27-like (RPS27L) in DNA repair has emerged by the findings showing the reduced expression of two double-strand break repair protein, RAD51 and protein kinase, DNA-activated, catalytic polypeptide (PRKDC) in conditions of RPS27L knockdown in colorectal cancer cells [63]. More recently, it has been reported that in lung cancer cells, RPS27L physically binds Fanconi anemia group D2 (FANCD2) and Fanconi anemia group I (FANCI), two proteins involved in DNA damage and repair. The inactivation of RPS27L impairs DNA interstrand cross-link repair by promoting the degradation of FANCD2 and FNCI through the p62-mediated autophagy-lysosome pathway [64].

### 3.4. Ribosome Free RPs Control Apoptosis

Among the several extra-ribosomal functions of RPs, one that is well documented is their involvement in the regulation of the apoptotic pathway. Here, we discuss some examples of RPs involved in this process.

In addition to its role in DNA repair, as discussed in the previous paragraph, uS3 also exhibits a pro-apoptotic function. Co-Ip experiments suggest that this apoptotic effect is due to the physical interaction between uS3 and tumor necrosis factor receptor type 1-associated death domain protein (TRADD). The pro-apoptotic signal mediated by uS3 seems to be executed through the activation of the caspase-8/caspase-3 cascade and c-Jun N-terminal kinase (JNK) pathway. The two functions of uS3—DNA repair and apoptosis—involve independent functional domains [65]. However, other studies show that uS3 silencing by siRNA triggers mitochondrial-mediated apoptosis in melanoma cell lines overexpressing uS3 [66]. In light of these findings, the potential role of uS3 in the apoptotic process needs to be still clarified.

Several studies have reported uS14 (S29) as an apoptosis-inducing agent in different human cancer cell lines. In laryngeal carcinoma cells, enhanced expression of uS14 induces p38 MAPK and JNK signaling, leading to the activation of apoptosis. It has been proposed that uS14 exerts its apoptotic function through the activation of both apoptotic pathways—death receptor-mediated and mitochondrial-mediated [67]. Although the proteomic analysis in cells overexpressing uS14 has confirmed the pro-apoptotic role of this CRP, its therapeutic efficacy in the treatment of laryngeal carcinoma needs to be confirmed by in vivo animal studies [67].

Our research group has identified uL3 as a pro-apoptotic factor involved in the induction of late apoptosis in p53-deleted colon cancer cells [68]. It functions as a regulator of oxidative stress response genes during acquired drug resistance. In p53-mutated drug-resistant lung cancer cells, uL3 expression is downregulated; its restoration re-sensitized the cells to the drug by regulating ROS levels, glutathione (GSH) content, glutamate release and cystine uptake. uL3 regulates the expression of stress-response genes, acting as a transcriptional repressor of solute carrier family 7 member 11 (xCT) and glutathione S-transferase alpha 1 (GST-α1) [37].

Zhang et al. have studied the effect of uS8 knockdown on glioblastoma cancer cells and demonstrated that uS8 silencing induces intrinsic mitochondrial apoptosis by modulation of B-cell lymphoma 2 (Bcl-2) protein levels [69].

In addition, knockdown of eS7 (S7) in ovarian cancer cells differentially regulates the expression of pro- and anti-apoptotic proteins, resulting in attenuated apoptosis. eS7 silencing is associated with the specific downregulation of pro-apoptotic factors such as p27^cip/kip^, BAX, Bcl-2 antagonist/killer (BAK), and Bcl2-associated agonist of cell death (BAD) and upregulation of anti-apoptotic factors Bcl-2 and B-cell lymphoma-extra-large (Bcl-xl). Although the relevance of eS7 in ovarian tumorigenesis migration and invasion pathways has been confirmed in vivo, further investigations are needed to define the clinical significance of eS7 in ovarian cancer [70].

Among the multiple regulators of the intrinsic apoptotic pathway, recent studies have reported the involvement of some MRPs [71].

mL41, also named BMRP (BCL-2 interacting mitochondrial ribosomal protein), contributes to p53 stability and suppresses the growth of cancer cells in nude mice, inducing p53-dependent apoptosis [55]. mL41 contains BCL-2 binding sites near its N-terminus. Since these sites are absent in the mature protein, the interaction of mL41 with BCL-2 and its apoptotic activity are likely to occur in the cytosol [72]. However, the mechanism underlying mL41’s pro-apoptotic role remains to be further investigated [55].

Controversial is the role of mL42 (MRPL42), also known as programmed cell death protein 9 (PCDP9), in the modulation of apoptosis. It has been demonstrated that mL42 triggers apoptosis by regulating multiple signaling involving Bcl-2, c-jun and JNK, whereas in glioma cells, mL42 silencing resulted in the activation of caspase-3/caspse-7 activity. Thus, the role of mL42 in the regulation of apoptosis remains to be more deeply investigated [73].

mS29 (MRPS29) was first discovered as a member of the death-associated protein (DAP) family and termed DAP3. It induces mitochondria-mediated apoptosis through the activation of p38, MAPK and JNK signaling in human laryngeal carcinoma cell line. Although other mechanisms of cell death involving mS29 have been found, its role in the apoptosis pathway remains to be clarified [74]. Moreover, the role of mS29 appears controversial, since its overexpression confers resistance to apoptosis in glioblastoma cells [75].

Finally, the depletion of bL35m (MRPL35) strongly increases ROS accumulation which, in turn, leads to DNA damage and cell cycle arrest at the G2/M phase. Hence, the higher ROS production acts on the mitochondria, disrupting the ΔΨm and inducing apoptosis and autophagy [76].

### 3.5. Ribosome Free RPs Are Involved in ER Stress and Autophagy

During tumorigenesis, protein demand is higher to sustain the uncontrolled cancer cell growth. The endoplasmic reticulum (ER) is an organelle responsible for the regulation of protein, lipid and steroid synthesis and calcium-dependent signaling. It plays a central role in controlling protein homeostasis and folding. The quality control systems of the ER regulate the trafficking of correctly folded proteins and target the misfolded ones for proteolysis simultaneously [77]. Under certain conditions such as cancer, the protein degradation process may be insufficient, resulting in the accumulation of the misfolded and unfolded proteins with consequent ER stress induction. As a response to a prolonged ER stress condition, the Unfolded Protein Response (UPR) signal transduction cascade, aiming to restore cellular homeostasis, is activated [77].

Emerging evidence highlights an interconnection between extra-ribosomal functions of RPs and UPR. In αβ T cell progenitors, eL22 (L22) silencing exacerbates ER stress and strongly activates two of the three ER stress pathways, i.e., protein kinase R (PKR)-like endoplasmic reticulum kinase (PERK) and Inositol-requiring enzyme 1 (IRE1α) signaling [78]. eL19 (L19) also activates the UPR, sensitizing breast cancer cells to ER stress-induced cell death, but the mechanism by which eL19 exerts this effect has not been fully elucidated [79]. More recently, transcriptomic RNA seq analysis of colon cancer cells devoid of p53 and stably silenced of uL3 has evidenced the activation of the UPR pathway, providing evidence of uL3 involvement in the regulation of this process [80,81].

The UPR and autophagy are interconnected and, recently, a link between the altered expression of some CRPs and autophagy modulation has emerged. Several stresses induced by mutations or deficiency of some CRPs may lead to autophagy induction in different cell types [81]. To date, in breast and ovarian cancer cell lines, the depletion of RPs such as uL10, RPLP1 and RPLP2 causes an increase in autophagic occurrence [82], whereas RPS27L silencing causes the blockage of this process via the mammalian target of rapamycin complex 1 (mTORC1) inactivation [83].

Recently, our research group has highlighted the role of uL3 as a negative regulator of autophagy in colon cancer. Analysis of the autophagic flux has demonstrated that uL3 overexpression markedly suppresses autophagic flux, which is associated with the decreased expression of protein components of autophagy, initiating the ULK complex [80,81]. Accordingly, in uL3-deleted colon cancer cells, the autophagic flux was most markedly increased. Of note, the restoration of uL3 in these cells is associated with the decrease in autophagy [80].

To the best of our knowledge, only a few data regarding the modulation of autophagy by MRPs are reported in the literature. Recently, Zhang et al. have demonstrated the role of bL35m (MRPL35) in autophagy occurrence. Specifically, bL35m knockdown leads to the upregulation of DNA damage-regulated autophagy modulator 1 (DRAM1) and autophagy-related 5 (ATG5) expression, both required for autophagy induction [76].

### 3.6. Ribosome Free RPs Affect Cell Migration

Cancer cell migration is essential for the promotion of tumor invasion from the primary site into adjacent and distant tissues, which is a primary cause of tumor recurrence.

Some studies have shown that RPs may play a role in the migration and invasion of malignant tumors. eL34 (L34), belonging to the L34E family of CRPs, is shown to be dysregulated in several types of tumors. This protein promotes the proliferation, migration and invasion of pancreatic and glioma cancer cells both in vitro and in vivo [84,85]. At the molecular level, the silencing of eL34 suppresses the proliferation and migration of glioma cells through inactivation of the JAK/STAT3 signaling pathway [85].

A crucial step of tumor invasion includes the degradation of the extracellular matrix (ECM) and the basement membrane (BM). Among the proteolytic enzymes produced by cancer cells and involved in the invasion, matrix metalloproteinases (MMPs) are zinc-dependent enzymes that participate in the degradation of ECM macromolecules. Enhanced expression of eS6 (S6) strongly increases the cell migration ability, upregulating MMP2 expression, while knockdown of eS6 reduces cell invasive capacity downregulating MMP9 and MMP2 expression [86]. The oncogenic and pro-metastatic role of eS6 has been also shown in esophageal squamous cell carcinoma cells as well as in ovarian cancer cells [87,88].

In human fibrosarcoma cells, uS3 strongly reduces cell invasive potential by blocking the ERK pathway and MMP-9 secretion through its interaction with nm23-H1, a known tumor cell metastasis suppressor in various cancers [89]. Some CRPs and MRPs, such as uL3 and mS40 (MRPS18-B), respectively, control cell migration affecting the epithelial–mesenchymal transition (EMT) process. Specifically, the low expression of uL3 is associated with EMT transition, which results in a more aggressive and invasive cancer phenotype [37], whereas the enhanced expression of mS40 leads to the induction of C-X-C Motif Chemokine Receptor 4 (CXCR4) expression and, consequently, the upregulation of twist-related protein 2 (TWIST2) and the repression of epithelial markers [90].

The oncogenicity of bS16m (MRPS16) and mL42 in the development of glioma has been recently demonstrated. In particular, the overexpression of bS16m promotes the migration and invasion of glioma cells by activating PI3K/Akt/Snail axis [71,91].

## 4. RPs as Biomarkers in Cancer Diagnosis

As already mentioned, several studies identify the alteration of ribosome biogenesis and function as key steps for the establishment of advantageous growth parameters of cancer cells [92]. It is now clear that the processes of ribosome biogenesis and translation can be envisaged as a platform in which some factors allow nodes of interconnection between metabolism, tumorigenesis and chemoresistance.

In this scenario, the study of alteration in the ribosome biogenesis process in cancer cells provides promising perspectives for the implementation of predictive biomarkers for early diagnosis, prognosis and therapy.

Loss of ribosome structural components causes a range of pathologically defined ribosomopathies, characterized by complex syndromes and increased insurgence of cancer [92], raising the question on how decreased levels of RPs increase malignancy. Somatic mutations in uL18 and uL16 have been described in T-cell acute lymphoblastic leukemia (T-ALL) [90]; mutations in uS19 (S15), uL15 (L27A) and eL22 have been described in 10–40% of multiple tumor types; and uL18 is also mutated in several cancers such as T-ALL like glioblastoma, melanoma, breast cancer and multiple myeloma [93].

Our group has contributed to the field, showing that uL3 is downregulated in p53-deleted colon cancer cells [36]. uL3 mutations are also present in Diamond-Blackfan anemia, a ribosomopathy characterized by bone marrow aplasia and increased hematological cancer [94]. Recent work from our lab has evaluated uL3 clinical significance in p53-deleted colon cancer tissues and demonstrated that uL3 expression decrease is associated with malignant progression and tumor grade and correlated with the development of resistance to different chemotherapeutic agents such as 5-FU and OHP [32,58]. In light of these observations, uL3 can be considered as a novel biomarker correlating with worse survival and resistance to 5-FU and/or OHP treatment in p53-deleted colon cancer. Our study highlights the importance of a therapy based on tumor biology rather than on histological criteria [95]. The study of individual tumor biology might enhance the efficacy of drugs, avoiding unnecessary drug-related toxicity in patients whose tumor will not respond.

Quantitative analysis of human prostate cancer tissues (CaP) has led to the identification of three RPs, i.e., eS19 (S19), eS21 (S21) and eS24 (S24), overexpressed in CaP patients [96]. Changes in eS19 and eS21 levels also correlated with high and low Gleason grade CaP, suggesting that these CRPs may be helpful markers for prognostic purposes. Furthermore, two of these three proteins, i.e., eS19 and eS24, showed different localization in malignant vs. non-malignant CaP tissues. Thus, the association of quantitative analysis and localization studies of these CRPs in CaP tissues may be useful for the diagnosis and/or prognosis of CaP.

Of interest, the efficacy of eS24 as a biomarker has been already reported for human colon carcinoma [96].

uS17 (S11) has been identified as a potential prognostic biomarker in hepatocellular carcinoma (HCC). In particular, a study conducted on a cohort of 182 patients has demonstrated that high uS17 levels were associated with shorter survival and recurrence-free survival after surgical resection [97].

uS17, uS9 (S16) and uS13 (S18) have been also identified as putative biomarkers for response to Topoisomerase II (TOP2) in the treatment of glioblastoma, the most malignant brain tumor in adults. Loss of uS17 is associated with acquired resistance to TOP2 poisons. It has been demonstrated that the expression of uS17 was necessary for the induction of apoptosis through apoptotic protease activating factor-1 (APAF1) protein [98].

The expression of several MRPs has also been found altered in different cancers, suggesting that, as with cytosolic RPs, MRPs could have a potential prognostic value [71]. In particular, in breast cancer, uL15m (MRPL15), uL13m (MPRL13) and mL54 (MPRL54) protein levels correlated with cancer recurrence, distant metastasis and prognosis. However, the specific mechanisms underlying the activity of these proteins in this cancer have yet to be explored.

In HCC, the decreased expression of uL13m is associated with the aggressive phenotype of liver cancer cells [71,99]. However, mS23 overproduction was found to induce HCC cell proliferation and to be associated with reduced survival of HCC patients [100]. Similar to mS23, the upregulation of mL66 (MRPS18-A) expression can promote the development of liver cancer [71,101].

Furthermore, some evidence suggests that several genes expressing MRPs including bS1m (MRPS28), uS2m (MRPS2), uL23m (MRPL23), uS12m (MRPS12), bL12m (MRPL12) and mS34 (MRPS34) may be potential biomarkers for the treatment of glioblastoma with Benzyl isothiocyanate (BITC) [102].

The relevance of mL38 (MRPL38) to cancer prognosis has been highlighted in ovarian cancer, by comparing the mitochondrial proteomic profile of human ovarian carcinoma cell lines with different metastatic potentials. mL38 was found to be more abundant in most metastatic cancer cell lines and, therefore, positively correlated with the aggressiveness of the tumor [74].

bL21m (MRPL21) and uL16m (MRPL16) have been identified as a potential prognostic marker in colorectal tumors [74].

Thus, the characterization and understanding of the abnormal expression of both cytosolic and mitochondrial RPs could provide a great tool in cancer diagnosis, prognosis and treatment outcomes.

## 5. RPs as Molecular Target in Cancer Treatment

A number of studies have demonstrated the altered expression of several individual RPs in different types of human cancers and the identification of new extra-ribosomal functions of some RPs have established these proteins as a novel class of oncogenic or tumor suppressor factors [27,28].

In prostate cancer, the expression of uS5 (S2) and eL19 is significantly elevated and some studies have identified these proteins as novel therapeutic targets. To date, the silencing of uS5 through a ribozyme-like oligonucleotide delivered locally or systemically seems to eradicate metastatic tumors in mice. In addition, Wang et al. have demonstrated that uS5 blocks pre-let-7a-1 miRNA processing, promoting the expression of oncogenes, such as Ras and c-Myc, and the transformation of primary prostate cell lines to a malignant phenotype. These suggest that silencing of uS5 may be a potential therapeutic strategy in prostate cancer treatment [103].

eL39 (L39) and eL32 (L32) are potential therapeutic targets for the treatment of breast cancer. It has been shown that gene silencing to shut off the function of these CRPs exerted anticancer effects [104,105].

Silencing of genes encoding specific CRPs, given their ubiquity, represents a strategy that may kill not only tumor cells but also healthy cells. Targeted therapy against r-proteins without adverse effects on normal cells is likely to prove challenging. In this regard, the opportunity to selectively target cancer cells by using nanoparticles carrying tags specific for receptors overexpressed by cancer cells may represent a valid and promising therapeutic strategy [106]. Depletion of eL39 by specific small interfering RNA (siRNA) packaged in liposome nanoparticles caused a significant tumor reduction in patient-derived cancer xenograft tumors as well as in combination with chemotherapy. At the molecular level, eL39 affects nitric oxide synthase signaling and hypoxia regulating inducible nitric oxide synthase (iNOS) and hypoxia-inducible factor 1 (HIF1) α. Silencing of eL32 using lentivirus (LV)-delivered siRNA strongly reduces cancer cell migration and invasion in vitro and in vivo [105].

Some findings have demonstrated that eL32 might be a promising therapeutic target for lung cancer; its overexpression was associated with poor prognosis in lung cancer patients. It has been shown that eL32 silencing affects ribosome biogenesis stress, causing the translocation of uL18 and uL5 from the nucleus to the nucleoplasm. Here, these proteins bind MDM2, leading to the accumulation of p53 that results in the inhibition of lung cancer proliferation [107].

eS6 is significantly upregulated in many cancers, including non-small cell lung cancer, esophagus squamous cell carcinoma, pancreatic neuroendocrine tumors and sarcoma. In particular, eS6 has been proposed as a therapeutic target for patients with ovarian cancer. Knockdown of eS6 by LV-small hairpin RNA (shRNA) remarkably inhibits the migration and invasion ability of ovarian cancer cells and induces G0/G1 phase arrest [88].

As with overexpression, loss of individual RPs is also associated with specific alterations in cellular phenotype.

uL3 is a key determinant in cellular stress response; as with ribosome free form, uL3 induces cell cycle arrest and apoptosis and inhibits autophagy [49,68,80,108]. The overexpression of uL3 increases the antitumoral effect of some anticancer drugs [50,109,110], whereas depletion of uL3 confers resistance to drugs such as 5-FU in p53-mutated lung and p53-deleted colon cancer cells [37,38,111,112].

**Figure 3:**
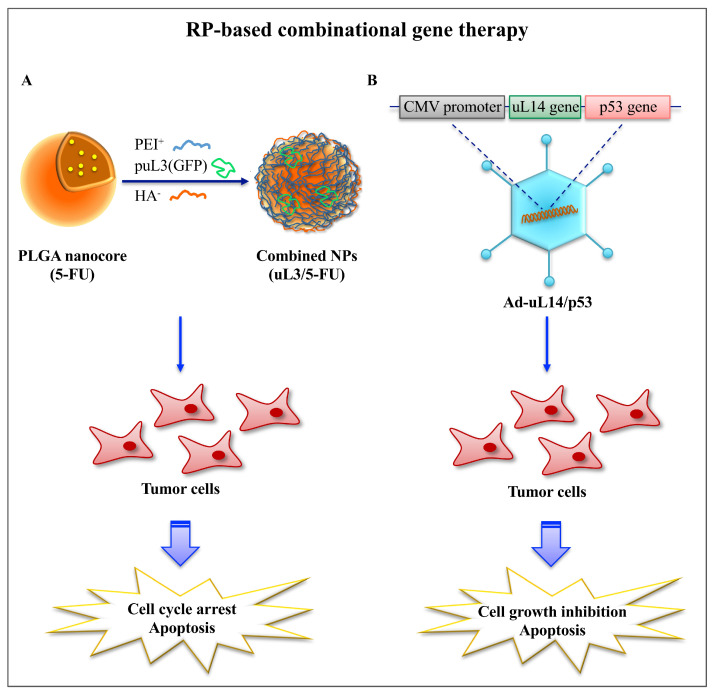
Strategies of RP-based gene therapy. (**A**) Polymeric nanoparticles with a core of PLGA and a polymer shell of HA and PEI as platform to deliver the 5-FU and the pro-apoptotic protein uL3. (**B**) Bicistronic adenovirus expressing both the uL14 and p53 genes (Ad-uL14/p53).

Restoration of uL3 protein level through transfection of uL3 expression vector re-sensitize the resistant cells to chemotherapy drugs by regulating ROS levels, GSH content, and cystine uptake [37]. Transfer of uL3 DNA along with 5-FU in tumors expressing low levels of uL3 protein by using polymeric nanoparticles based on a core of poly(lactic-co-glycolic) acid (PLGA) and a polymer shell of Hyaluronan (HA) and Polyethyleneimine (PEI) resulted in the induction of apoptosis and accumulation of cells in sub-G1, consistent with functional uL3 activity (Figure 3) [36,113].

With a similar approach, the co-transduction of uL14 enhances the therapeutic efficacy of adenoviral-mediated p53 in suppressing the proliferation of p53-mutated cancer cells (Figure 3). Ectopic uL14 protein stabilizes p53, leading to its accumulation in cells accompanied with increased expression levels of the p53 downstream target genes MDM2 and p21 in vitro and in vivo [114]. Furthermore, eL41 (L41) induces the degradation of the activating transcription factor 4 (ATF4), a major regulator of tumor cell survival, contributing to sensitize tumor cells to chemotherapy [115].

## Figures and Tables

**Table 1:** Dedicated CRP chaperons.

	Human New (old) CRP	Chaperon	Refs
Large ribosomal subunit	uL3 (L3)	Rrb1	[11]
	uL16 (L10)	Sqt1	[11]
	uL4 (L4)	Acl4	[12]
	uL14 (L23)	Bcp1	[13]
	uL18 (L5), uL5 (L11)	Syo1	[14]
Small ribosomal subunit	uS3 (S3)	Yar1	[15]
	eS26 (S26)	Tsr2	[11]
	uS11 (S14)	Fap7	[16]

**Table 2:** RPs regulate cell cycle progression.

Molecular Target	Mammalian New (old) RP	Refs
Cyclin B1/CDK1	uL10m (MRPL10)	[47]
p21	uL3 (L3)	[48,49]
p27	uS15 (S13)	[50]
p21, CDK1, pAkt	uS8 (S15A)	[51,52]
E2F1	eL21 (L21)	[53]
p53	MRPS36 mL41 (MRPL41)	[54,55]

## Data Availability

Not applicable.
